# In-line near-infrared analysis of milk coupled with machine learning methods for the daily prediction of blood metabolic profile in dairy cattle

**DOI:** 10.1038/s41598-022-11799-0

**Published:** 2022-05-16

**Authors:** Diana Giannuzzi, Lucio Flavio Macedo Mota, Sara Pegolo, Luigi Gallo, Stefano Schiavon, Franco Tagliapietra, Gil Katz, David Fainboym, Andrea Minuti, Erminio Trevisi, Alessio Cecchinato

**Affiliations:** 1 Department of Agronomy, Food, Natural Resources, Animals and Environment (DAFNAE), University of Padua, 35020 Legnaro (PD), Italy; 2 Afimilk Ltd., 1514800 Kibbutz Afikim, Israel; 3 Department of Animal Science, Food and Nutrition (DIANA) and the Romeo and Enrica Invernizzi Research Center for Sustainable Dairy Production (CREI), Faculty of Agricultural, Food and Environmental Sciences, Università Cattolica del Sacro Cuore, 29122 Piacenza, Italy

**Keywords:** Computational biology and bioinformatics, Metabolism, Animal behaviour, Animal physiology, Lasers, LEDs and light sources

## Abstract

Precision livestock farming technologies are used to monitor animal health and welfare parameters continuously and in real time in order to optimize nutrition and productivity and to detect health issues at an early stage. The possibility of predicting blood metabolites from milk samples obtained during routine milking by means of infrared spectroscopy has become increasingly attractive. We developed, for the first time, prediction equations for a set of blood metabolites using diverse machine learning methods and milk near-infrared spectra collected by the AfiLab instrument. Our dataset was obtained from 385 Holstein Friesian dairy cows. Stacking ensemble and multi-layer feedforward artificial neural network outperformed the other machine learning methods tested, with a reduction in the root mean square error of between 3 and 6% in most blood parameters. We obtained moderate correlations (*r*) between the observed and predicted phenotypes for γ-glutamyl transferase (*r* = 0.58), alkaline phosphatase (0.54), haptoglobin (0.66), globulins (0.61), total reactive oxygen metabolites (0.60) and thiol groups (0.57). The AfiLab instrument has strong potential but may not yet be ready to predict the metabolic stress of dairy cows in practice. Further research is needed to find out methods that allow an improvement in accuracy of prediction equations.

## Introduction

Health monitoring of dairy herds is pivotal to improving their health and welfare and to attain greater efficiency and sustainability in farming. Nutritional imbalances, dietary deficiencies or improper management, especially during the transition and early lactation phases, can generate a range of health disorders, which are generally categorized as metabolic diseases, and include ketosis, hepatic lipidosis, hypocalcemia and hypomagnesemia^1^. Typically, as milk production increases and herds become larger, the incidence of metabolic diseases increases, becoming a major source of economic losses^2,3^.

Blood biochemistry is commonly used as part of a diagnostic evaluation to confirm the suspected disease, assess prognosis, control the progression of disease, and appraise the effectiveness of treatments^4^. On the other hand, analysis of blood biochemical constituents, known as metabolic profiling, is a well-established test that, in association with animal, diet and management assessments, helps determine disease risk in clinically healthy dairy cows, rather than diagnose disease^4,5^. Serum metabolic profiling is of particular interest for identifying subclinical disorders, which are highly prevalent and have serious consequences for the cows’ welfare and production levels^6–8^. Detection of individuals with alterations to their blood parameters could allow early nutritional or management interventions to be delivered to avert the onset of overt affections. Anyhow, blood sampling remains a labor-intensive, invasive procedure that causes the cows distress^9^. Furthermore, laboratory analyses of comprehensive metabolic panels are costly and time-consuming^10,11^, limiting their application at the farm level. In contrast, collecting milk samples is noninvasive and can be easily incorporated into standard milking procedures. Moreover, milk composition reflects the metabolic status of cows, in that deviations from metabolic homeostasis are manifested by alterations in milk composition^12,13^. Infrared spectroscopy uses infrared light to scan milk samples and detect the presence of specific chemical bonds, producing an absorption profile comprising the absorbance values for individual infrared light wavenumbers associated with milk components^14^. The use of milk infrared spectroscopy as an alternative source of information on animal health status has therefore become increasingly attractive^12,15^. Indeed, the use of minimally invasive sensors and technology able to collect a large amount of data in order to understand and predict the status of animals is fundamental to improving sustainable production systems and precision livestock farming^16^.

In dairy herds, Fourier-transform mid-infrared (2500 to 25,000 nm; FTIR) spectra of milk are widely used as a cost-effective means of assessing raw milk composition^17^. Diverse studies have pointed out the ability of FTIR in predicting metabolites dissolved in milk, such as acetone, β-hydroxybutyric acid (BHBA) and citrate^18^. More ambitiously, in recent years numerous efforts have been made to indirectly predict blood metabolites, such as glucose, IGFs, non-esterified fatty acids (NEFA), BHBA, urea, proteins and minerals using milk FTIR spectra at the individual cow level^8,9,19^. Although the milk FTIR predictions in these studies did not provide precise quantification of blood components, they nonetheless highlighted the potential of FTIR analysis to screen for high or low values of individual blood metabolites or combinations of them as metabolic status indicators. However, FTIR spectroscopy requires a large laboratory, so that while milk samples can be taken from each cow once or twice per month for herd level analyses, to date the technology is not suitable for on-farm daily management.

Near-infrared (350 to 2500 nm; NIR) spectra, on the other hand, appear to be just as reliable as FTIR spectra for analyzing raw milk composition and cheese-making traits^20–22^. The visible to low-NIR range (350 to 1000 nm) seems to be suitable for automated in-line analysis of milk, as the optical sensors are less expensive^23^. Other advantages include rapidity, simplicity, simultaneous nondestructive measurements, lower sensitivity to environmental noise, and greater robustness compared with far-NIR (1000 to 2500 nm) and FTIR measurements^24,25^. Moreover, fine milk components such as fatty acids, that are known to represent a fingerprint of the cow’s nutritional and metabolic status, have been reported to be satisfactorily predictable using NIR instruments^26^. AfiLab (Afimilk, Kibbutz Afikim, Israel) is an in-line NIR spectrometric milk analyzer that can be installed in the milking parlor to provide real-time measurements during milking sessions^27^. As pointed out in a recent review^15^, the possibility of predicting blood components from the milk of individual cows with a continuous system could be an important step forward in evaluating the health status of cows and identifying individual susceptibility to metabolic disorders.

To the best of our knowledge, no equations to indirectly predict blood parameters from milk NIR spectra have ever been developed. Previous studies on phenotype prediction of milk traits from milk NIR spectra have mainly used partial least squares (PLS) regression models^23,28^. However, several new machine learning methods, including deep and ensemble learning can now be applied, providing greater flexibility in modeling complex associations and improving prediction accuracy compared with PLS methods^29–31^.

The objectives of the present study were twofold. First, we assessed the feasibility of using daily milk NIR spectra, through the AfiLab real-time milk analyzer, to predict a broad set of 29 hematochemical parameters, such as metabolites related to energy metabolism, liver function/hepatic damage, oxidative stress, inflammation/innate immunity, and minerals in clinically healthy, high-yielding Holstein Friesian dairy cows. Second, we explored the inner structure of the NIR spectra predictions, identifying for each hematochemical indicator the best prediction model and the relevance of individual wavelengths in explaining the variations in all the traits of concern.

## Results and discussion

### Blood metabolic profile of cows

Descriptive statistics of blood metabolite concentrations within our cohort are reported in Table 1. As all the cows involved in the study were clinically healthy, the range of variability in the data is representative of a physiological condition. Although the cows did not manifest clinical disease, the high variability in some blood biomarkers means we cannot exclude the presence in certain individuals of subclinical conditions, an expected finding in a large population^32^.

**Table 1:** Descriptive statistics for hematochemical parameters in all cows involved in the study.

Hematochemical parameters^a^	N	mean	SD	P1^b^	P99^b^
Hematocrit, l/l	381	0.31	0.03	0.25	0.37
**Energy-related metabolites**					
Glucose, mmol/l	385	4.47	0.30	3.74	5.26
Cholesterol, mmol/l	384	4.97	1.19	2.04	7.77
NEFA, mmol/l	384	0.13	0.17	0.02	0.53
BHBA, mmol/l	385	0.52	0.20	0.25	1.29
Urea, mmol/l	385	6.56	1.04	4.09	9.07
Creatinine, µmol/l	385	81.15	5.59	71.76	93.39
**Liver function/hepatic damage**					
AST, U/l	385	101.10	24.97	68.35	179.10
GGT, U/l	385	26.86	8.26	14.63	52.80
BILt, µmol/l	385	2.21	1.13	0.55	6.58
Albumin, g/l	385	37.08	2.27	29.19	41.45
ALP, U/l	385	67.08	19.51	33.55	124.33
PON, U/ml	385	104.56	19.22	58.53	154.53
**Oxidative stress metabolites**					
ROMt, mgH_2_O_2_/100 ml	385	12.61	3.24	5.89	23.24
AOPP, µmol/l	385	47.77	9.15	29.68	72.18
FRAP, µmol/l	384	208.11	62.58	125.83	315.00
SHp, µmol/l	355	389.88	51.43	271.32	515.92
**Inflammation/innate immunity**					
Ceruloplasmin, µmol/l	385	1.78	0.61	0.75	3.76
PROTt, g/l	385	81.21	4.83	71.71	95.08
Globulins, g/l	385	44.12	5.40	36.19	60.96
Haptoglobin, g/l	385	0.37	0.33	0.10	1.50
Myeloperoxidase, U/l	385	457.69	72.90	284.72	671.20
**Minerals**					
Calcium, mmol/l	385	2.52	0.11	2.21	2.78
Phosphorus, mmol/l	385	2.01	0.34	1.23	2.86
Magnesium, mmol/l	385	1.00	0.10	0.74	2.24
Sodium, mmol/l	385	143.25	2.79	135.86	148.39
Potassium, mmol/l	385	4.21	0.36	3.47	5.22
Chlorine, mmol/l	385	103.03	2.26	97.06	109.79
Zinc, µmol/l	385	11.45	2.24	6.36	17.10

^a^*NEFA* non-esterified fatty acids, *BHBA* β-hydroxybutyric acid, *AST* aspartate aminotransferase, *GGT* γ-glutamyl transferase, *BILt* total bilirubin, *ALP* alkaline phosphatase, *PON* paraoxonase, *ROMt* total reactive oxygen metabolites, *AOPP* advanced oxidation protein products, *FRAP* ferric reducing antioxidant power, *SHp* thiolic groups, *PROTt* total proteins.

^b^P1 and P99 represents 1 and 99% of the trait quantile in percentage.

Looking at the major blood indicators of metabolic impairment in dairy cows and their well-documented thresholds, we observed a certain degree of alteration in serum proteins, with 11% of cows presenting with elevated globulins concentrations (> 50 g/l), and 2% with low albumin concentrations (< 30 g/l)^33^. We found 1.5% of cows (n = 6) with BHBA concentrations equal to or greater than 1.2 mmol/l^34^, whereas fewer than 1% of cows (n = 3) had elevated NEFA concentrations, in accordance with a threshold of ≥ 0.70 mmol/l^35^. Of the six hyperketotic cows, one had a concurrent elevated concentration of NEFA. The low prevalence of hyperketonemia may be due to the fact that the cows in our study ranged across all lactation stages (between 3 and 504 days in milk [DIM]), with only 1.5% (n = 6) in the first 5d postpartum, when the peak incidence of hyperketonemia occurs^7^. Regarding urea concentration, 43% of cows exceeded the threshold of ≥ 6.78 mmol/l^36^. The level of blood urea reflects the effects of dietary intake of crude protein and its digestive utilization, milk protein secretion, body protein turnover and nitrogen (N) urinary excretion^37^. High-producing dairy cattle are commonly fed diets with crude protein levels exceeding 16% to ensure maximum milk output^38^, as was the case in the study herd (i.e. 16.5% dietary crude protein with an average milk yield of 33.9 kg/days). The significance of high values of blood or milk urea in high-yielding cows is controversial; several studies reported negative effects on the conception rate in dairy cows^39,40^, while others found no such effects^41,42^. Nevertheless, if protein fed to ruminants exceeds microbial needs, it degrades to ammonia, is absorbed into the blood, converted to urea in the liver and excreted in the urine. Impairment of the N balance may result in laminitis and metabolic dysfunction^43^. Conversely, no animals had serum urea concentrations below the optimal range (< 1.7 mmol/l)^44^. Less than 1% of cows were suspected of having hypomagnesemia (< 0.70 mmol/l) or hypocalcemia (< 2.0 mmol/l)^45^.

### Model performances

Regarding the performances of the machine learning models, the prediction accuracy obtained through random 10-10-fold cross-validation (CV) was greater than through PLS regression for all the blood traits except for BHBA, which had a correlation coefficient (*r*) of 0.63 with PLS. In fact, the *r* values for the PLS regression predictions were < 0.25 for 23 of the 29 blood traits, making them unsuitable for further analysis (data not in Table). It is worth noting that we applied a completely independent CV (random or leave-one-batch-out), so none of the records in the training sets was present in the validation sets for all folds generated. Applying the leave-one-batch-out CV, where each batch is constituted by a different sampling date, accuracy dropped by between 5 and 75% compared with 10-fold CV for all traits except for globulins and hematocrit, where accuracy increased slightly, by 3% and 9%, respectively (Table 2). This increases the intrinsic source variability in blood metabolite measurements, which are subject to critical fluctuations in relation to physiological variations, environmental conditions, sampling procedure and timing^46,47^. The leave-one-batch-out CV technique reflects a more conservative scenario, where there is less dependence between the training and validation sets, which reduces the predictive ability of the models compared with the 10-fold scheme^48,49^. It is worth nothing that in our dataset there is no batch dependence bias, so the different batches may under or overperform the prediction depending on the blood metabolites.

**Table 2:** Near-infrared AfiLab milk prediction performance considering the systematic effect of days in milk and parity through different cross-validation scheme for hematochemical parameters using machine learning.

Hematochemical parameters^a^	*r*		RMSE		Slope	
	10-fold	Batch-out	10-fold	Batch-out	10-fold	Batch-out
Hematocrit, l/l (log_10_)	0.30	0.33	0.01	1.23	− 8.02	16.74
**Energy-related metabolites**						
Glucose, mmol/l	0.61	0.42	4.18	0.29	0.94	0.93
Cholesterol, mmol/l	0.65	0.39	0.90	1.13	0.95	0.66
NEFA, mmol/l (log_10_)	0.44	0.16	0.01	0.01	0.71	− 0.05
BHBA, mmol/l (log_10_)	0.54	0.50	0.12	0.13	0.85	0.66
Urea, mmol/l	0.62	0.54	0.82	0.88	0.92	0.94
Creatinine, µmol/l (log_10_)	0.42	0.36	0.03	0.03	0.73	0.67
**Liver function/hepatic damage**						
AST, U/l (log_10_)	0.45	0.37	0.08	0.09	0.91	0.91
GGT, U/l (log_10_)	0.58	0.46	0.10	0.10	0.91	0.68
BILt, µmol/l (log_10_)	0.42	0.15	0.16	0.18	0.73	0.34
Albumin, g/l	0.58	0.28	1.92	2.28	0.72	0.31
ALP, U/l	0.54	0.40	16.45	17.11	0.88	0.68
PON, U/ml	0.31	0.17	18.82	20.40	0.58	0.29
**Oxidative stress metabolites**						
ROMt, mgH_2_O_2_/100 ml	0.60	0.39	2.59	2.91	0.95	0.51
AOPP, µmol/l	0.44	0.11	8.30	8.98	0.72	0.12
FRAP, µmol/l (log_10_)	0.56	0.15	0.08	0.11	0.92	− 0.20
SHp, µmol/l	0.57	0.33	42.79	53.29	0.92	0.76
**Inflammation/innate immunity**						
Ceruloplasmin, µmol/l	0.46	0.35	0.56	0.58	0.74	0.55
PROTt, g/l	0.57	0.54	4.03	4.13	0.82	0.84
Globulins, g/l	0.61	0.63	4.18	4.30	0.94	0.87
Haptoglobin, g/l	0.66	0.32	0.01	0.01	0.91	0.21
Myeloperoxidase, U/l	0.47	0.42	65.60	64.06	0.81	0.79
**Minerals**						
Calcium, mmol/l	0.36	0.26	0.11	0.12	0.65	0.59
Phosphorus, mmol/l	0.39	0.11	0.32	0.41	0.68	0.32
Magnesium, mmol/l	0.45	0.34	0.10	0.11	0.71	0.72
Sodium, mmol/l	0.65	0.51	2.18	2.40	0.86	0.83
Potassium, mmol/l	0.32	0.22	0.36	0.40	0.61	0.44
Chlorine, mmol/l	0.30	0.14	2.26	2.37	0.46	0.18
Zinc, µmol/l	0.59	0.47	1.88	2.26	0.87	0.74

All the presented results are the average of the different folds (10 for the 10-fold and 5 for the leave-one-batch-out).

RMSE = root mean square error.

^a^*NEFA* non-esterified fatty acids, *BHBA* β-hydroxybutyric acid, *AST* aspartate aminotransferase, *GGT* γ-glutamyl transferase, *BILt* total bilirubin, *ALP* alkaline phosphatase, *PON* paraoxonase, *ROMt* total reactive oxygen metabolites, *AOPP* advanced oxidation protein products, *FRAP* ferric reducing antioxidant power, *SHp* thiolic groups, *PROTt* total proteins.

Because no previous studies have been conducted on the prediction of blood parameters from NIR spectra captured from milk using the AfiLab system, and due to the peculiarity of the data structure of each blood trait, we applied an automatic machine learning (autoML) method that used different penalized regression models (ridge regression [RR], least absolute shrinkage and selection operator [LASSO], elastic net [EN]), machine learning techniques (random forest [RF], gradient boosting machine [GBM], and multi-layer feedforward artificial neural network [ANN]), and stacking ensemble model, a combination of them to find the best model without assuming any prior knowledge. Of the different machine learning methods tested, the stacking ensemble and ANN were those with the highest predictive ability for most of the blood metabolite-related traits, as shown in Table 3 and Supplementary Table S1. Indeed, we obtained a reduction in the root mean square error (RMSE) of between 3 and 6% for both the stacking ensemble and ANN compared with the other models tested. Moreover, in the models that outperformed the others, there was consistency between the two CV schemes in all blood metabolites, with the exception of advanced oxidation protein products (AOPP) and minerals (Supplementary Table S1). On the one hand, stacking uses a learn-to-learn approach, whereby different algorithms are used as base learners, and the models with greater prediction accuracy are combined in a final prediction model with appropriate penalization, which provides more accurate predictions^50^. On the other hand, ANN has the ability to self-learn relationships from labeled data and generalize to unlabeled conditions, producing high performances on low variability traits^51^. A recent study tested ANN and found its performance to be accurate in predicting BHBA from milk FTIR spectra^30^.

**Table 3:** Best machine learning approaches through different cross-validation (CV) scheme for hematochemical parameters expressed as frequencies (how many times each model is the best model for a given CV scheme for each hematochemical parameter prediction).

Machine learning algorithm	CV scheme	Frequencies (%)	Hematochemical parameters^a^
Stacking ensemble	10-fold	48.4	ALP, AOPP, cholesterol, ceruloplasmin, globulins, BHBA, BILt, MPO, phosphorus, paraoxonase, ROMt, SHp, urea, zinc
	Batch-out	41.4	ALP, cholesterol, ceruloplasmin, globulins, BHBA, BILt, MPO, paraoxonase, potassium, ROMt, SHp, urea
Multi-layer feedforward artificial neural network	10-fold	41.4	Albumin, AST, chlorine, potassium, haptoglobin, creatinine, FRAP, GGT, NEFA, magnesium, sodium, PROTt
	Batch-out	44.8	Albumin, AST, AOPP, chlorine, haptoglobin, creatinine, FRAP, GGT, NEFA, magnesium, sodium, phosphorus, PROTt
Distributed random forest	10-fold	3.4	Calcium
	Batch-out	0	–
Gradient boosting machine	10-fold	3.4	Hematocrit
	Batch-out	6.9	Calcium, hematocrit
Elastic net	10-fold	3.4	Glucose
	Batch-out	6.9	Glucose, zinc

^a^*NEFA* non-esterified fatty acids, *BHBA* β-hydroxybutyric acid, *AST* aspartate aminotransferase, *GGT* γ-glutamyl transferase, *BILt* total bilirubin, *ALP* alkaline phosphatase, *PON* paraoxonase, *ROMt* total reactive oxygen metabolites, *AOPP* advanced oxidation protein products, *FRAP* ferric reducing antioxidant power, *SHp* thiolic groups, *PROTt* total proteins.

As pointed out by Eskildsen et al.^52^, the predictive ability of a spectrum equation can be affected by individual factors (e.g. breed, parity, DIM) and herd-related factors (e.g. feed, management). Indeed, the inclusion of DIM and parity effects has been reported to enhance the robustness of prediction equations^22,53^. Here, to examine the potential of NIR analysis, we built prediction equations on the basis of a single breed and a single herd, and included in the models the main sources of variation in lactating cows (i.e. DIM and parity), as these data are provided by the management software of the Afimilk system installed in the farm. It is worth noting that, unlike other studies that used mid-infrared devices within milk recording schemes, in our study we developed—for the first time—tailored NIR AfiLab prediction equations that could be used to check blood indicators on a daily basis at the farm level without the need for milk sampling or any type of processing, making the detection of individuals with putative metabolic dysfunctions faster and easier and enabling timely intervention.

### Relationships between light emitting diodes and blood traits

**Figure 1:**
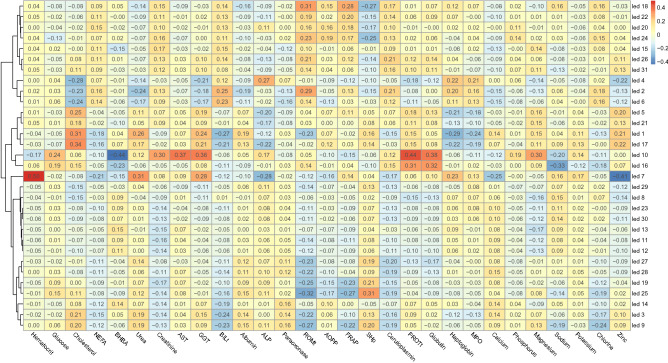
Pearson correlations heatmap between light emitting diodes (LEDs) information and blood metabolites in all Holstein–Friesian cows involved in the trial. On the left side, unsupervised hierarchical clustering of LEDs is showed as a dendrogram. *NEFA* non-esterified fatty acids, *BHBA* β-hydroxybutyric acid, *AST* aspartate aminotransferase, *GGT* γ-glutamyl transferase, *BILt* total bilirubin, *ALP* alkaline phosphatase, *PON* paraoxonase, *ROMt* total reactive oxygen metabolites, *AOPP* advanced oxidation protein products, *FRAP* ferric reducing antioxidant power, *SHp* thiolic groups, *PROTt* total proteins. Graphics have been created using the *corrplot* R package within the R software v. 3.6.3 (www.r-project.org).

The AfiLab spectrophotometer consists of an array of light emitting diodes (LEDs) at 32 discreet wavelengths^54,55^. As milk is a complex fluid, being a suspension of emulsified butterfat globules and casein micelles in a water-based solution, the dynamic interactions between light and matter in it are highly nonlinear. Overall, we found low correlations between each LED and the blood metabolites (*r* < 0.50); the red regime, green regime, and water absorption NIR lines (LEDs 7, 10 and 16) appeared to contain the most informative wavelengths for blood traits (Fig. 1). As expected, we observed agreement in LED relationships between specific and derived blood traits (i.e. globulins from total proteins).

### Predictive ability, meaning and biological link with milk of all blood traits

**Figure 2:**
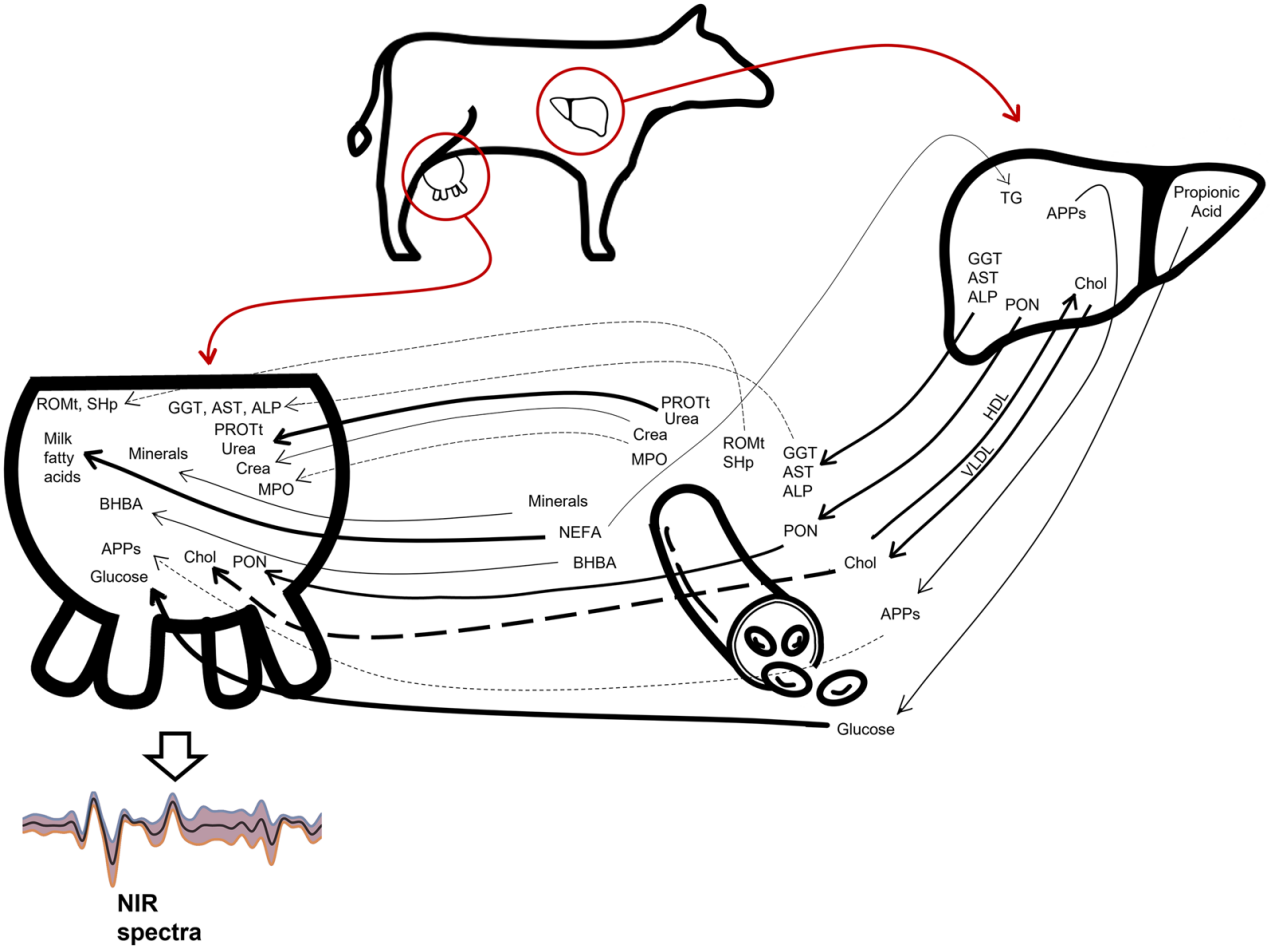
Origin of substances present in mammary gland of dairy cows captured with near-infrared (NIR) spectra and relationships with blood stream and liver. The thick arrows represent gross transfer between compartments during lactation; in the mammary gland, the metabolites involved in these processes are mainly derived from blood flow. The thin arrows denote minor fluxes. Dashed lines represent fluxes of metabolites that are partly originated from the blood stream and partly secreted directly by mammary gland. During lactation, circulating non esterified fatty acids (NEFA) are regularly incorporated into milk fat; circulating NEFA in excess are stored in the liver as triglycerides (TG). Cholesterol (Chol) in the mammary gland originates from blood uptake and, to a certain extent, from local synthesis in the mammary tissue. Propionic acid represents the substrate for gluconeogenesis: the mammary gland absorbs large amounts of glucose from blood flow to synthetize lactose, enhance viability and proliferation of the mammary cells, and supply energy for synthesis of milk components. Acute phase proteins (APPs), including haptoglobin and ceruloplasmin, are mainly synthetized in the liver and are able to pass through the blood-mammary barrier in case of systemic inflammatory conditions or, when local processes are active, they could be self-produced by the mammary epithelium. *ALP* alkaline phosphatase, *AST* aspartate aminotransferase, *BHBA* β-hydroxybutyric acid, *Crea* creatinine, *GGT* γ-glutamyl transferase, *HDL* high density lipoproteins, *MPO* myeloperoxidase, *PON* paraoxonase, *PROTt* total proteins, *ROMt* total reactive oxygen metabolites, *SHp* thiolic groups, *VLDL* very low density lipoproteins.

We explored which—if any—of the blood metabolites in a metabolic profile could be reliably predicted from milk NIR spectra. The fitting statistics of the prediction models of blood metabolites are reported in Table 2. The predictive ability of a spectrum equation is trait dependent, and it relies on the covariance structures between the investigated traits and milk composition^52^. As our findings show, blood traits that are well known to be secreted in the udder obtained the highest* r* values (Fig. 2). Since this is the first attempt to predict a wide range of serum metabolites from milk NIR spectra using the AfiLab system, we are unable to draw parallels with previous literature.

Among the energy-related metabolites, glucose, total cholesterol, and urea attained an *r* higher than 0.6 and BHBA 0.54. For this category of traits, the most informative LED was the 16 (Supplementary Fig. S1). A recent study on milk NIR spectra obtained greater accuracy (*r* = 0.71) than we did in the prediction of urea, but the reference values in that study were collected on the same matrix as the predicted values (milk urea)^56^. To our knowledge, BHBA and NEFA have not previously been estimated from milk NIR spectra, but when predicted from milk FTIR spectra, they exhibited high (*r* > 0.70) prediction accuracies, leading to this technique being proposed as a monitoring tool for detecting hyperketonemia^30,57,58^. In the current study, NEFA prediction equations had weak reliability; the unsatisfactory estimation was probably due to poor variability in the samples and the non-normal distribution of the data, which included a very low proportion of high values. Moreover, logarithm transformation did not significantly improve the predictive ability (data not in Table). For BHBA, we obtained a moderate *r* value of CV with both the random (0.54) and leave-one-batch-out (0.50) schemes, but the low number of samples with a BHBA higher than 1.2 mmol/l means our equations were unable to correctly categorize ketotic animals. A higher number of hyperketotic cows therefore need to be included in the analysis to confirm the potential usefulness of our equations in screening dairy cows for subclinical ketosis. However, given the nature of circulant BHBA, which drops after feeding^59^, even if the equations are poorly calibrated two or three milking sessions a day might be a better way of screening for subclinical ketosis in cows than daily blood sampling at random times.

Within the category of blood indicators of liver function and hepatic damage, albumin and γ-glutamyl transferase (GGT) had an *r* of 0.58, and alkaline phosphatase (ALP) an *r* of 0.54. Again, LED 7 and LED 10 were the wavelengths with the greatest explanatory capacity (Supplementary Fig. S2). Hepatic enzymes are physiologically present in cows’ milk as a result of spontaneous diffusion of low molecular weight enzymes from plasma or active secretion by the mammary gland epithelium, or they are released after disruption of somatic cells, most often leukocytes. In the case of GGT and ALP, their activity has been reported to be much greater in milk than in blood serum, and they not only correlate with increased plasma activity, but also with stage of lactation, seasonality, milk yield, and mammary gland health^60,61^. In any case, a relationship between energy metabolism and aspartate aminotransferase- glutamate oxaloacetate transaminase (AST), ALP and GGT activity in the mammary gland has been shown, with a strong correlation between blood and milk hepatic enzymes, and with mild degrees of hepatic lesions showing an increase in enzymes in both matrices^61–63^. Therefore, the potential to properly predict hepatic enzymes from milk on a daily basis deserves further investigation.

Blood indicators of oxidative stress were found to be reasonably predictable from milk NIR spectra, with total reactive oxygen metabolites (ROMt), ferric reducing antioxidant power (FRAP) and thiol groups (SHp) showing *r* values between 0.57 and 0.60. The LED 25 was the most informative for this group of traits (Supplementary Fig. S3). Parameters that measure oxidative stress conditions have not previously been predicted using infrared spectroscopy techniques. Plasma levels of ROMt indicate free radical production; conversely, total SHp of plasma are considered a significant element in the extracellular antioxidant defense system against oxidative stress and can be used to describe antioxidant potential in dairy cows^64^.Total reactive oxygen metabolites and SHp are present in milk^65^, and SHp can be detected by NIR spectra wavelengths^66^. Information on the relationship between the blood levels of oxidant/antioxidant metabolites and milk concentrations is scarce, but suggests there is a relationship between oxidative status and innate immune status in blood and milk^67,68^. The ability to predict the oxidative status of cows from milk NIR spectra might shed light on the overall stress conditions of each animal, but further research is needed to understand whether the levels of oxidative stress metabolites in milk are representative of blood concentrations.

With regard to blood indicators of inflammation and innate immunity, prediction accuracies were low for ceruloplasmin and myeloperoxidase, but were better for haptoglobin, total proteins and globulins (*r* = 0.66, 0.57, and 0.61, respectively). The LEDs providing most information on these traits are 7, 10, 13 and 16 (Supplementary Fig. S4). Haptoglobin is an acute-phase protein and is a sensitive indicator of local or systemic inflammation, which can occur prior to the development of typical signs of inflammation (e.g. inflammatory leucogram)^69^. It is known to be diffused from blood into the milk, but it is also produced endogenously by milk leukocytes and epithelial cells during mammary gland processes^70^. For these reasons, haptoglobin has been proposed has an inflammatory indicator in dairy cows^70^. Animals with signs of clinical or subclinical mastitis were excluded from this study, so the levels of haptoglobin detected might be suggestive of blood diffusion. Further studies including greater numbers of individuals might lead to predicted milk haptoglobin being used as an indicator of the metabolic status of dairy cows without ongoing mammary gland disorders. Total proteins, globulins and albumin in blood, on the other hand, are indicators of both physiological (e.g. age, breed, parity, stage of lactation, nutrition, climate, season) and pathological variations in dairy cows^71,72^. The presence of blood-derived proteins in milk is well documented, although the relationships in active and passive transfers are complex^73^. Notably, repeated measures have been highlighted as a successful strategy for correctly evaluating their concentrations^71^. As such, the potential to accurately evaluate serum protein with a continuous system, avoiding invasive, time-consuming blood sampling, becomes much more relevant.

Among blood minerals, only sodium and zinc appeared to be predictable from milk NIR spectra, their *r* values being 0.65 and 0.59, respectively. Sodium was predicted using mainly LED 16, zinc using LEDs 7, 10 and 16 (Supplementary Fig. S5). These results are not unexpected, for two main reasons. First, the minerals in this dataset exhibited extremely low variability compared to other blood metabolites (Table 1). Moreover, the minerals exhibiting greater variability had higher *r* values, supporting the hypothesis that traits with a wider range of variability provide better prediction models. Second, minerals do not have specific band absorptions in the near-infrared spectrum, so their predictability from NIR spectra might be related to their occurrence in organic compounds, such as proteins or organic molecules^74^, or to the effect of the element on the water absorption band^75^, as already shown in other food matrices^76,77^. In this study, we predicted blood metabolites from milk spectra. Whereas a large proportion of reference values for minerals are in their ionized forms, such as 50% for calcium and 70% for magnesium, the majority of minerals in milk are in organic compounds: two-thirds of calcium is bound in organic compounds within the colloidal phase, more than 50% of total phosphorus is bound to casein micelles, and only 16% of magnesium is present as free ions^78^. Furthermore, inorganic forms such as inorganic phosphorus (representing 80% of milk phosphorus) are difficult to detect using NIR spectra^74^. Sodium, instead, is found mainly as free ions in both blood and milk^78^, as is zinc, which is present prevalently in its ionized form^79^. Furthermore, the amounts of sodium and zinc are markedly affected by inflammatory conditions, in both blood and milk. We can speculate that this similarity of chemical form in the two matrices, coupled with the alteration they bring about in the water region of the spectrum^80^, might explain better predictive ability of our equations compared to the rest of the minerals^76^.

Overall, the models we developed for predicting blood traits were found to have moderate predictive ability (*r* < 0.70) according to recommended thresholds^81^ and compared to predictions of milk blood metabolites from FTIR spectra^8,9,57^. Although it has major advantages over FTIR, including rapidity of acquisition, cost-effectiveness, and continuous recording, NIR spectrometry uses light scattering principles to measure particle size, which makes it difficult to capture the nonlinear diffusive scattering produced by milk, a complex, heterogeneous matrix^25^. Near-infrared spectra are highly influenced by the presence or absence of water^15^, and raw milk has a high water content^23^. Despite all this, while FTIR is difficult to implement in the in-line process due to operational difficulties at the individual cow level and the need for repeated reference measurements, NIR has been successfully installed in in-line instruments (such as the AfiLab milk analyzer). With the implementation of prediction equations in in-line devices, it is possible to check the metabolic conditions of cows multiple times daily, allowing for real-time intervention to improve health status. From a managerial perspective, the availability of real-time metabolic indicators is a crucial step forward in the monitoring of subclinical diseases. Improvements to the prediction equations of milk NIR spectra installed in in-line devices could be the answer to the problem of monitoring the health of individual cows.

In conclusion, milk is a complex matrix, whose composition mirrors the metabolic status of the cow. By means of infrared spectroscopy, a wealth of information in the form of absorption profiles can be extracted on a daily basis using a non-invasive technique and stored for subsequent individual health evaluations. Moreover, the development of prediction equations based on milk NIR spectra using machine learning methods yielded better results than using traditional techniques (i.e. PLS), especially for hepatic enzymes and inflammatory indicators. We showed that prediction equations based on NIR spectra are not only able to predict components directly traceable in milk, but can also provide information on indirect indicators, such as blood metabolites, offering insights into the health status of the cow. The prediction equations for haptoglobin and hepatic enzymes, and for oxidative stress parameters, in particular, yielded promising results.

Nevertheless, in view of applying these equations in daily practice to monitor the health status and management of cows, further research is needed to better dissect the relationships between the NIR spectra and the milk matrix, improve accuracy, and identify the most informative NIR wavelengths for blood indicators of metabolic distress. Moreover, a larger sample that includes animals with clinical disease, thereby widening the population variability and improving the accuracy and repeatability of the predictions, is required to improve the accuracy of the prediction equations.

## Methods

### Study design and field data

This study is part of a broader project (PROH-DAIRY) funded by the Ministero degli Affari Esteri e della Cooperazione Internazionale (MAECI) within the Italy-Israel R&D Cooperation Program aimed at developing new precision livestock breeding tools as a contribution towards One Health in the Italian and Israeli dairy chains. To accomplish the objectives of the project and develop prediction equations able to predict diverse traits from milk using infrared technology, an AfiLab real-time milk analyzer (Afimilk, Kibbutz Afikim, Israel) was installed de novo in the milking parlor of the study farm to collect milk infrared spectra. The Afifarm management program (Afimilk, Kibbutz Afikim, Israel) was used for daily herd management and for storing the data, including the AfiLab spectra per cow per milking sessions (morning and evening).

The farm participating in the study is located in the production area of Grana Padano Protected Designation of Origin (PDO) hard cheese in Piacenza province (northwestern Italy). It holds a commercial herd comprising 965 lactating Holstein Friesian cows with an average milk yield around 9500 to 10,000 kg per cow per year. For this study, milk spectra data were obtained from 385 cows in their first to fifth parity from October 2019 to December 2020. The cows’ number of days in milk covered the entire lactation period (3 to 504 DIM). They were housed in free stalls and were fed on total mixed rations based mainly on corn silage, sorghum silage, and concentrates (Supplementary Table S2). Drinking water was available in automatic water bowls, and the cows were milked twice daily. Management information (e.g. feeding, DIM, parity, health status) was available, but was not experimentally manipulated.

Approval for the study was granted by the ethical committee of the Organismo Preposto al Benessere degli Animali (OPBA; Organization responsible for animal welfare) of the Università Cattolica del Sacro Cuore, and by the Italian Ministry of Health (protocol number 510/2019-PR of 19/07/2019). The study was carried out following the recommendations of the ARRIVE guidelines.

### Blood sampling

Animals with clinical signs of disease or undergoing medical treatment were excluded from the trial. Blood samples (5 ml) were collected from the jugular vein of 385 animals and placed in vacuum tubes containing 150 USP units of lithium heparin (Vacumed; FL Medical, Torreglia, Padua, Italy). Blood sampling of each cow was carried out after the morning milking and before feeding from September 2019 to February 2020 (9 different herd/dates).

### NIR spectra collection and preprocessing

The AfiLab system is a spectrophotometer that consists of an array of 32 discreet wavelengths in the range of visible to low-NIR (350 nm to 1000 nm) based on LEDs as previously described by Schmilovitch et al.^25^. During the morning milking, NIR spectra were obtained from individual cows using the AfiLab system (Afimilk, Kibbutz Afikim, Israel; internal control), which collects infrared information from each 200 ml of milk flowing through the machine, then averages the information from each cow in each milking session. The AfiLab system is routinely calibrated automatically once a month to avoid accumulating bias in the infrared collection system.

**Figure 3:**
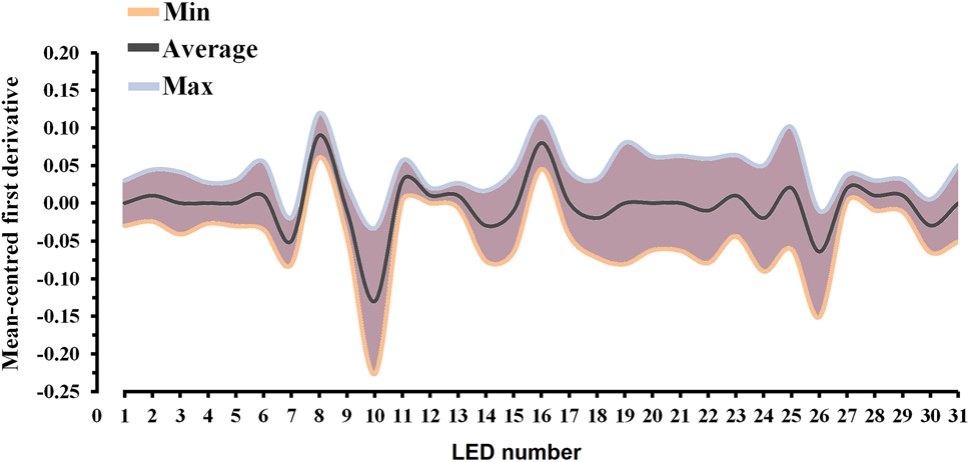
The average value for AfiLab milk spectra (solid black line represents the average, the purple region represents values inside mean ± 3*SD, the blue line represents the maximum value, and orange line the minimum value) that belonged to the considered Holstein–Friesian cows’ population (n = 385). *LED* light emitting diode.

The milk spectra acquired from each animal were preprocessed by the first derivative^82^, which was then normalized using a Standard Normal Variate equation $$\left[ {SVN_{i} = {\raise0.7ex\hbox{${x_{i} - \overline{{x_{i} }} }$} \!\mathord{\left/ {\vphantom {{x_{i} - \overline{{x_{i} }} } {s_{i} }}}\right.\kern-\nulldelimiterspace} \!\lower0.7ex\hbox{${s_{i} }$}}} \right]$$ (Fig. 3); this procedure is used to remove the baseline variations that can occur over time. Quality control of infrared spectra to remove possible outliers was performed combining principal component analysis and Mahalanobis distance, using a significance level of 5%^83^; three animals were removed from the subsequent analysis.

### Reference blood metabolic profile analysis and preprocessing

Once collected, the blood samples were kept on ice until centrifugation (Hettich Universal 16R Centrifuge, 3500 g, 16 min, 6 °C) and were processed within two hours of collection. Hematocrit was determined using a small fraction of the sampled blood (ALC Centrifugette 4203, 15,300 g, 12 min). The plasma obtained from centrifugation was stored at − 20 °C until analysis. An ILAB 650 (Instrumentation Laboratory, Bedford, MA) clinical auto-analyzer was used to determine the concentrations of glucose, NEFA, BHBA, urea, creatinine, calcium, phosphorus, magnesium, sodium, potassium, chlorine, zinc, AST, GGT, ALP, total proteins, haptoglobin, ceruloplasmin, albumin, total bilirubin, cholesterol and globulins, according to Calamari et al.^4^; ROMt, FRAP and paraoxonase, according to Bionaz et al.^32^; SHp, according to Minuti et al.^84^.; myeloperoxidase, according to Bradley et al.^85^.; and AOPP, according to Hanasand et al.^86^.

Mathematical treatments were applied to the blood metabolites prior to model development. The original values of hematocrit, NEFA, BHBA, AST, GGT, creatinine, total bilirubin, haptoglobin and FRAP had strongly skewed distributions with lower values over-represented, which could affect prediction accuracy^18^. After having tested some other transformation procedures, such as square root and natural logaritmic ones, we applied a logarithmic transformation (base-10), which gave the best improvement in reducing skewness and normalizing distribution (Supplementary Fig. S6).

### Prediction analysis and cross-validation scenarios

The performance of the infrared prediction models for blood metabolites were evaluated with 10-fold and leave-one-batch-out CV strategies. For the 10-fold CV, the dataset was split randomly into ten non-overlapping folds; in each iteration nine of the folds were used as the training set, and the remaining fold was assigned to the validation set. This process was repeated ten times, with each fold used once as the validation set. For the leave-one-batch-out CV, the dataset was randomly split on the basis of blood sampling date (i.e. into five batches); the training models thus comprised four batches, and one batch was assigned to the validation set. This process was repeated five times with each batch assigned once to the validation set.

### Statistical analysis

Blood metabolites were predicted using the automatic machine learning (autoML) algorithm of the *h2o* R package (https://github.com/h2oai/h2o-3), which automatically trains and adjusts the models. Within the *h2o.automl* function, fixed parameters were a maximum of 100 models and random combinations of the hyperparameters over 45 min. For each individual, models considered the AfiLab infrared data along with the on-farm information related to DIM and parity. To find the model with the best prediction accuracy, the autoML was run using three machine learning models (RF, GBM, and ANN), three penalized regression models (RR, LASSO, and EN), and combining the models with the best predictions using a stacking ensemble.

#### Penalized regression

This approach for phenotypic prediction uses LASSO, RR or EN, which is a combination of LASSO (λ_1_ regularization term) and RR penalties (λ_2_ regularization term), providing a balance between the two methods. Ridge regression uses the λ_2_ penalization in proportion to the sum of the squares of the regression coefficients in order to shrink the variables making lower contributions to close to zero, while LASSO considers λ_1_ penalization using the sum of the absolute values of the coefficients to shrink the regression coefficients making lower contributions towards zero, and performing variable selection to reduce model complexity. Elastic net regression considers both the λ_1_ and λ_2_ norm to effectively shrink the coefficients and set some of them to zero.

Random Forest and GBM are regression tree approaches to learning that combine individual trees through bootstrap aggregation (RF) or boosting (GBM). The RF algorithm fits different regression trees and ultimately combines them in the final predictive model^87^. The GBM fits a prediction model that converts weak learning into strong learning, adding models sequentially to the previous weak model to reduce variation and bias in the model^88^. Multi-layer feedforward artificial neural network, a deep learning technique, transforms the input information non-linearly through multiple hidden layers (neural network) before making the final prediction; the number of hidden layers defines the depth of the neural network, while the number of neurons in the layers defines its width.

To identify the best combination of hyperparameters of the penalized regression (RR, LASSO and EN) and machine learning (RF, GBM and ANN) techniques, a random search was carried out of the main parameters of each model using the default values of the AutoML function in the *h2o* R package (https://docs.h2o.ai/h2o/latest-stable/h2o-docs/automl.html). Stacking ensemble uses the concept of learn-to-learn, and makes predictions using different, previously-trained base learners (penalized regression and machine learning), and combines the best models in the final predictive model^89^. The base learner combination from the stacking ensemble uses a generalized linear model with a lambda search.

The predictive ability of the models was assessed by Pearson’s correlations (*r*) between the observed and predicted phenotypes, and the RMSE. The slope of the linear regression of the observed and predicted values in each model and CV strategy for the blood metabolite traits evaluated was used to assess the model’s unbiasedness. Each cow was used only once in each loop of the 10-fold CV in order to avoid having the same cow in both the test and training datasets at the same time but with different lactation orders. One loop ended when each sample had been used exactly once in testing. Each CV loop was repeated 500 times for each machine learning algorithm.

To compare the models’ performances with those using a traditional method, prediction equations for blood metabolic profiles were also developed using PLS regression implemented in the *pls* R package^90^, and model performance was evaluated with random 10-fold CV.

## Supplementary Information

Supplementary Information.

## Data Availability

The data that support the findings of this study are deposited with Afimilk Ltd., and access is restricted as they were used under license for the current study and are therefore not publicly available. However, they can be obtained from the authors upon reasonable request and with the permission of Afimilk Ltd.

## Code availability

The in-house code developed to perform the study is available from the authors upon request.
