# Supplementary material for: In-line near-infrared analysis of milk coupled with machine learning methods for the daily prediction of blood metabolic profile in dairy cattle
Source: Sci Rep. 2022 May 16;12:8058. doi: 10.1038/s41598-022-11799-0 (PMC9110744; doi:10.1038/s41598-022-11799-0)
Supplement: Supplementary file 1 — Supplementary Information. [file 41598_2022_11799_MOESM1_ESM.pdf]

In-line near-infrared analysis of milk coupled with machine learning methods for the daily prediction of blood metabolic profile in dairy cattle

Diana Giannuzzi, Lucio Flavio Macedo Mota, Sara Pegolo, Luigi Gallo, Stefano Schiavon, Franco Tagliapietra, Gil Katz, David Fainboym, Andrea Minuti, Erminio Trevisi, Alessio Cecchinato

Supplementary Table S1. Features of the best prediction machine learning models through different cross-validation (CV) scheme for hematochemical parameters.

Hematochemical parameters[1]	CV scheme	algorithm[2]	mean residual deviance	RMSE[2]	training time (ms)	predict time per row (ms)
Hematocrit, l/l (log_{10})	10-fold	GBM	0	0.0012	22579	0.006
	batch-out	GBM	0	0.0012	9107	0.0069
Energy-related metabolites						
Glucose, mmol/l	10-fold	EN	0.0638	0.2527	1946	0.0947
	batch-out	EN	0.0619	0.2488	71	0.0019
Cholesterol, mmol/l	10-fold	Stacking Ensemble	0.7249	0.8514	3069	1.6485
	batch-out	Stacking Ensemble	0.7221	0.8498	114	0.6336
NEFA, mmol/l (log_{10})	10-fold	ANN	0	0.0061	16100	0.2214
	batch-out	ANN	0	0.0035	24007	0.1688
BHBA, mmol/l (log_{10})	10-fold	Stacking Ensemble	0.0132	0.1149	284	0.5984
	batch-out	Stacking Ensemble	0.0122	0.1107	198	0.4728
UREA, mmol/l	10-fold	Stacking Ensemble	0.5798	0.7614	944	0.9694
	batch-out	Stacking Ensemble	0.5996	0.7743	8451	0.319
Creatinine µmol/l (log_{10})	10-fold	ANN	5.00E-04	0.0232	13086	0.364
	batch-out	ANN	5.00E-04	0.0227	15874	0.0294
Liver function/hepatic damage						
AST, U/l (log_{10})	10-fold	ANN	0.0051	0.0716	33603	0.0841
	batch-out	ANN	0.0051	0.0715	81690	0.0984
GGT, U/l (log_{10})	10-fold	ANN	0.0074	0.0863	15645	0.1113
	batch-out	ANN	0.0075	0.0866	26625	0.1741
BILt, µmol/l (log_{10})	10-fold	Stacking Ensemble	0.0199	0.141	2422	0.3787
	batch-out	Stacking Ensemble	0.0199	0.1412	14374	0.7685
Albumin, g/l	10-fold	ANN	3.0697	1.7521	33324	0.1772
	batch-out	ANN	3.2449	1.8014	65756	0.3047
ALP, U/L	10-fold	Stacking Ensemble	235.1727	15.3353	1690	3.188
	batch-out	Stacking Ensemble	250.2976	15.8208	36112	1.6698
PON, U/ml	10-fold	Stacking Ensemble	282.9373	16.8207	432	2.7044
	batch-out	Stacking Ensemble	304.3423	17.4454	2107	3.6665

Oxidative stress metabolites						
ROMt, $mgH_2O_2/100ml$	10-fold	Stacking Ensemble	5.9653	2.4424	5850	3.7587
	batch-out	Stacking Ensemble	5.6472	2.3764	7204	0.7171
AOPP, µmol/l	10-fold	Stacking Ensemble	54.5978	7.389	3669	1.1892
	batch-out	ANN	53.5267	7.3162	49087	0.9065
FRAP, µmol/l (log_{10})	10-fold	ANN	0.0051	0.0716	11722	0.0277
	batch-out	ANN	0.0048	0.0696	48271	0.0137
SHp, µmol/l	10-fold	Stacking Ensemble	1487.9639	38.5741	603	2.76
	batch-out	Stacking Ensemble	1538.8045	39.2276	3983	0.2364
Inflammation/innate immunity						
Ceruloplasmin, µmol/l	10-fold	Stacking Ensemble	0.2395	0.4894	4441	1.2962
	batch-out	Stacking Ensemble	0.251	0.501	486	0.6617
PROTt, g/l	10-fold	ANN	13.4938	3.6734	22476	0.1552
	batch-out	ANN	13.5838	3.6856	30496	0.1765
Globulins, g/l	10-fold	Stacking Ensemble	13.9478	3.7347	5990	6.4494
	batch-out	Stacking Ensemble	15.8444	3.9805	7587	5.2884
Haptoglobin, g/l (log_{10})	10-fold	ANN	1.00E-04	0.0098	21535	0.4366
	batch-out	ANN	1.00E-04	0.0107	43341	0.089
Myeloperoxidase, U/l	10-fold	Stacking Ensemble	3661.5752	60.511	153	0.1861
	batch-out	Stacking Ensemble	3499.3	59.1549	5653	1.8324
Minerals						
Calcium, mmol/l	10-fold	DRF	0.0096	0.0981	18186	0.1332
	batch-out	GBM	0.0098	0.0988	18919	0.1503
Phosphorus, mmol/l	10-fold	Stacking Ensemble	0.0853	0.2921	382	4.7368
	batch-out	ANN	0.0852	0.2918	13614	0.011
Magnesium, mmol/l	10-fold	ANN	0.0073	0.0856	13640	0.0277
	batch-out	ANN	0.0076	0.0872	55918	0.0967
Sodium, mmol/l	10-fold	ANN	3.9399	1.9849	29843	0.0502
	batch-out	ANN	3.5722	1.89	16348	0.0551
Potassium, mmol/l	10-fold	ANN	0.1071	0.3273	22654	0.1723
	batch-out	Stacking Ensemble	0.1087	0.3297	4704	1.2326
Chlorine, mmol/l	10-fold	ANN	3.6865	1.92	37496	0.1135
	batch-out	ANN	3.775	1.9429	28556	0.3988
Zinc, µmol/l	10-fold	Stacking Ensemble	2.8003	1.6734	286	0.5254
	batch-out	EN	2.8361	1.6841	19547	0.0875

[1]NEFA = non-esterified fatty acids; BHBA = β-hydroxybutyric acid; AST = aspartate aminotransferase; GGT = γ-glutamyl transferase; BILt = total bilirubin; ALP = alkaline phosphatase; PON = paraoxonase; ROMt =total reactive oxygen metabolites; AOPP = advanced oxidation of protein products; FRAP = ferric reducing antioxidant power; SHp = thiolic groups; PROTt = total proteins.
[2]GBM = Gradient Boosting Machine; EN = Elastic Net; ANN = multi-layer feedforward Artificial Neural Network; DRF = Distributed Random Forest.

Supplementary Table S2. Diet composition (% of DM) during lactation and dry period of the herd.

	Lactation	Dry
Corn silage	12.42	-
Sorghum silage	24.6	26.61
Wheat silage	-	-
Alfalfa hay	20.98	-
Ryegrass hay	2.12	47.15
Straw	-	14.14
Corn grain ground	12.62	-
Barley grain ground	8.41	-
Soybean meal	12.1	5.53
Sunflower meal	4.34	5.78
Soybean	-	-
Wheat midds	-	-
Hydrogenated fat	0.78	-
Minerals and vitamin supplements[1]	1.63	0.79
Nutrient composition:		
UFL (U/kg DM)	0.97	0.78
ENl (Mcal/kg DM)	1.55	1.31
Crude protein (% DM)	16.50	12.50
Metabolizable protein (% DM)	10.50	8.27
NSC (% DM)	36.80	19.00
NDF (% DM)	35.80	56.00

[1]During the dry period dairy cows received 70 g/d of a supplement contained 42.9% Ca_2PO_4; 28.6% urea; 14.3% MgO; 7.1% NaCl; 7.1% mineral vitamin supplement composited to provide 100000 UI of vitamin A, 10000 IU of vitamin D, 500 mg of vitamin E, 100 mg of Mn, 300 mg of Zn, 50 mg of Cu, 5 mg of I, 1 mg of Co, 3 mg of Se. During the lactation dairy cows received 300 g/d of a supplement contained, 27.5% $NaHCO_3$, 20% $CaCO_3$, 20% $CaHPO_4$; 7% MgO; 2% NaCl; 10% mineral vitamin supplement composited to provide, 150000 UI of vitamin A, 15000 IU of vitamin D, 1000 mg of vitamin E, 100 mg of vitamin K, 100 mg of vitamin H1 50 mg of vitamin B1, 0.5 mg of vitamin B12, 500 mg of vitamin PP, 4000 mg of choline, 700 mg of Mn, 1200 mg of Zn, 200 mg of Cu, 20 mg of I, 2 mg of Co, 4 mg Se.

Supplementary Figure S1. Variable importance values (%) for machine learning considering Afilab near infrared spectra, days in milk and parity for energy-related blood metabolites and hematocrit. CV = cross-validation method; LED = light emitting diode.

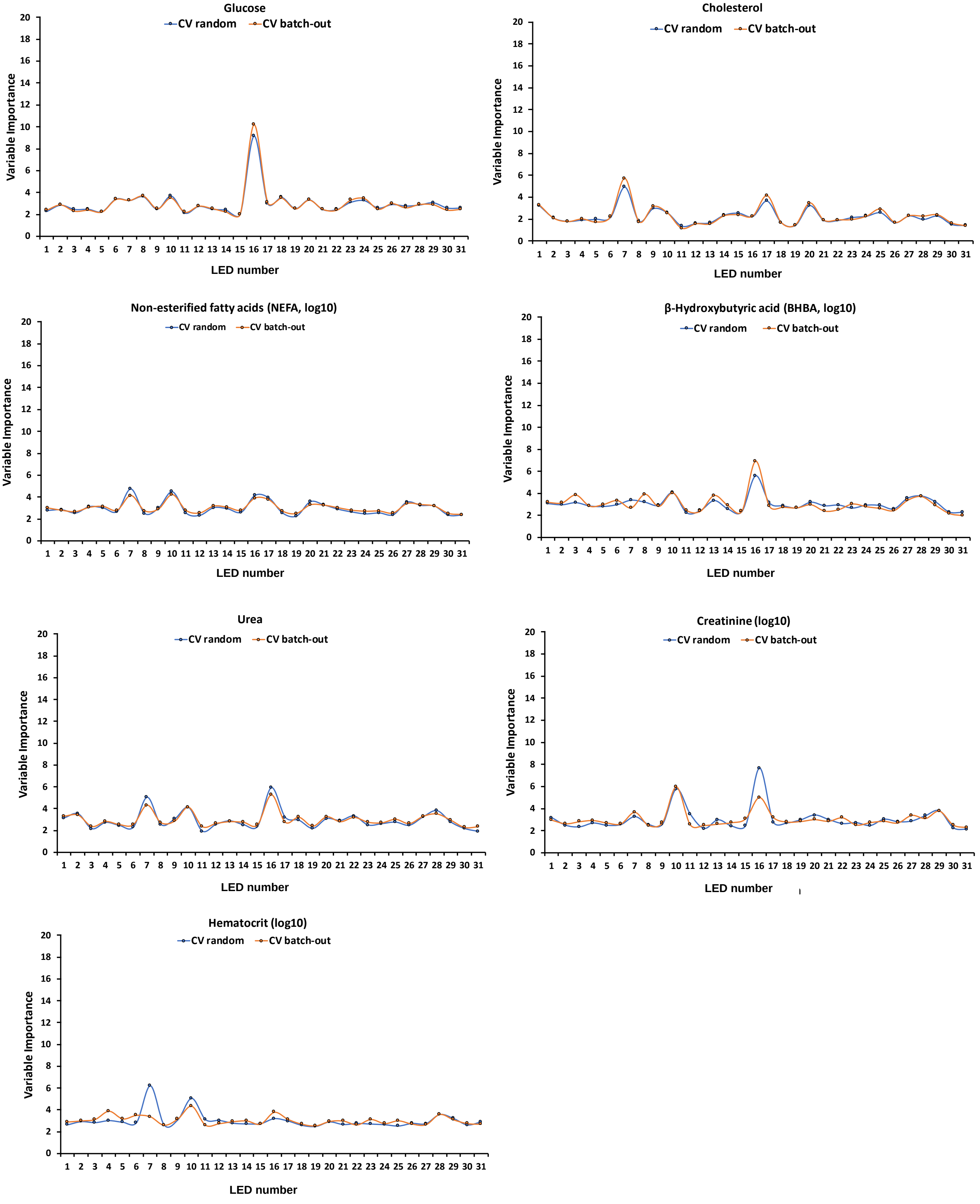

Supplementary Figure S2. Variable importance values (%) for machine learning considering NIR-AfiLab infrared, days in milk and parity for liver function/hepatic damage blood metabolites. CV = cross-validation method; LED = light emitting diode.

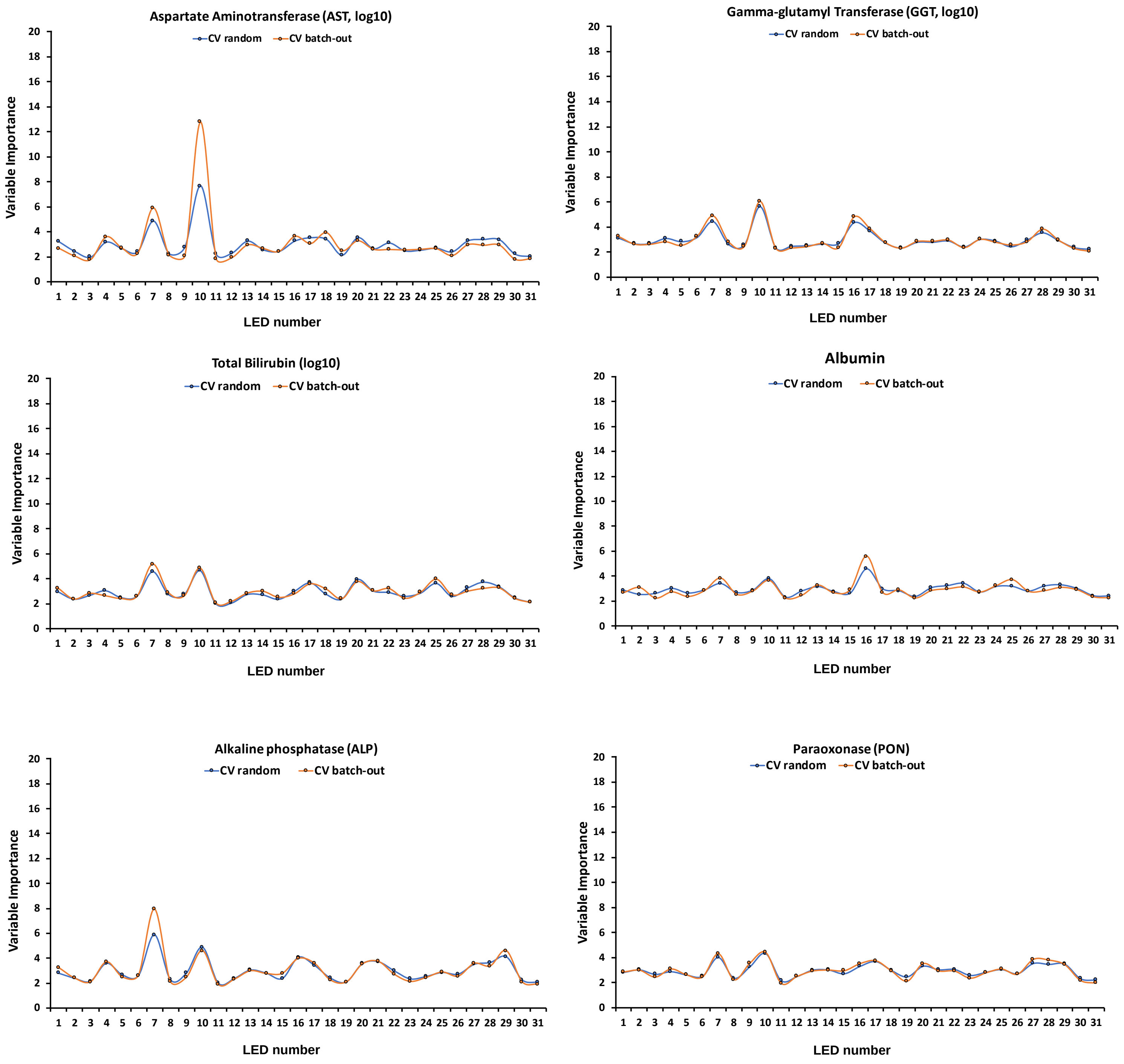

Supplementary Figure S3. Variable importance values (%) for machine learning considering NIR-Afilab infrared, days in milk and parity for oxidative stress blood metabolites. CV = cross-validation method; LED = light emitting diode.

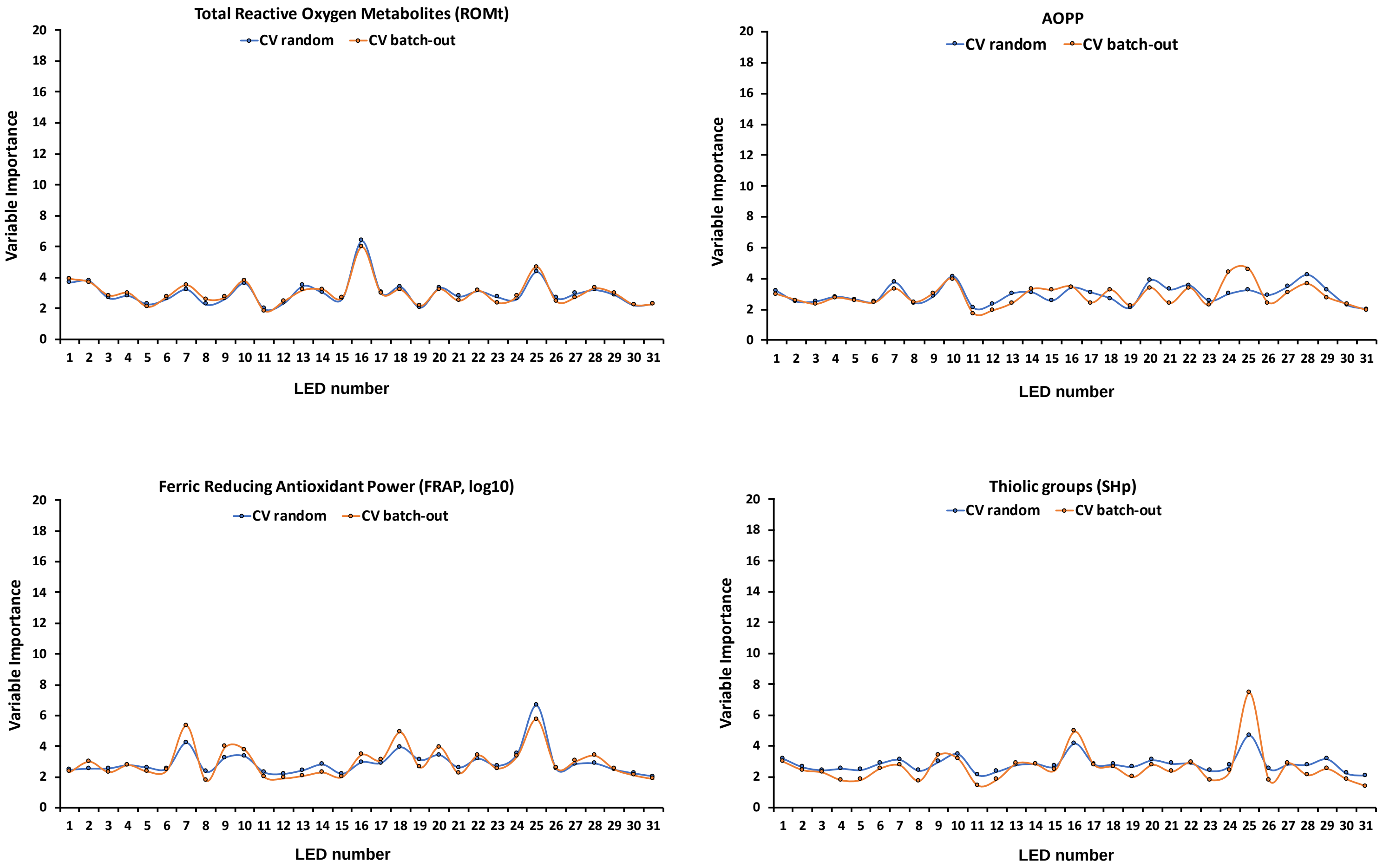

Supplementary Figure S4. Variable importance values (%) for machine learning considering NIR-Afilab infrared, days in milk and parity for inflammation/ innate immunity blood metabolites. CV = cross-validation method; LED = light emitting diode.

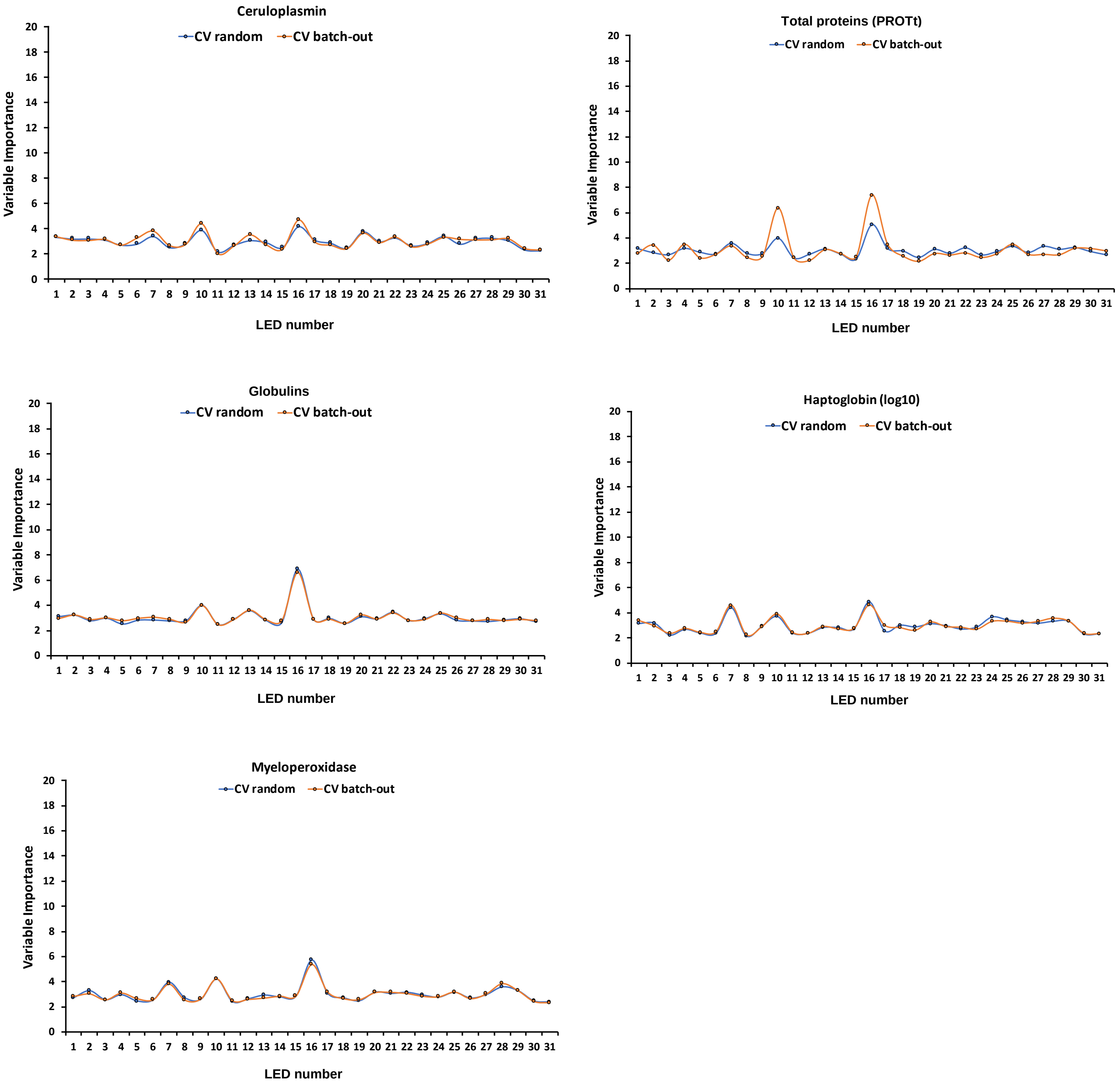

Supplementary Figure S5. Variable importance values (%) for machine learning considering NIR-Afilab infrared, days in milk and parity for blood minerals. CV = cross-validation method; LED = light emitting diode.

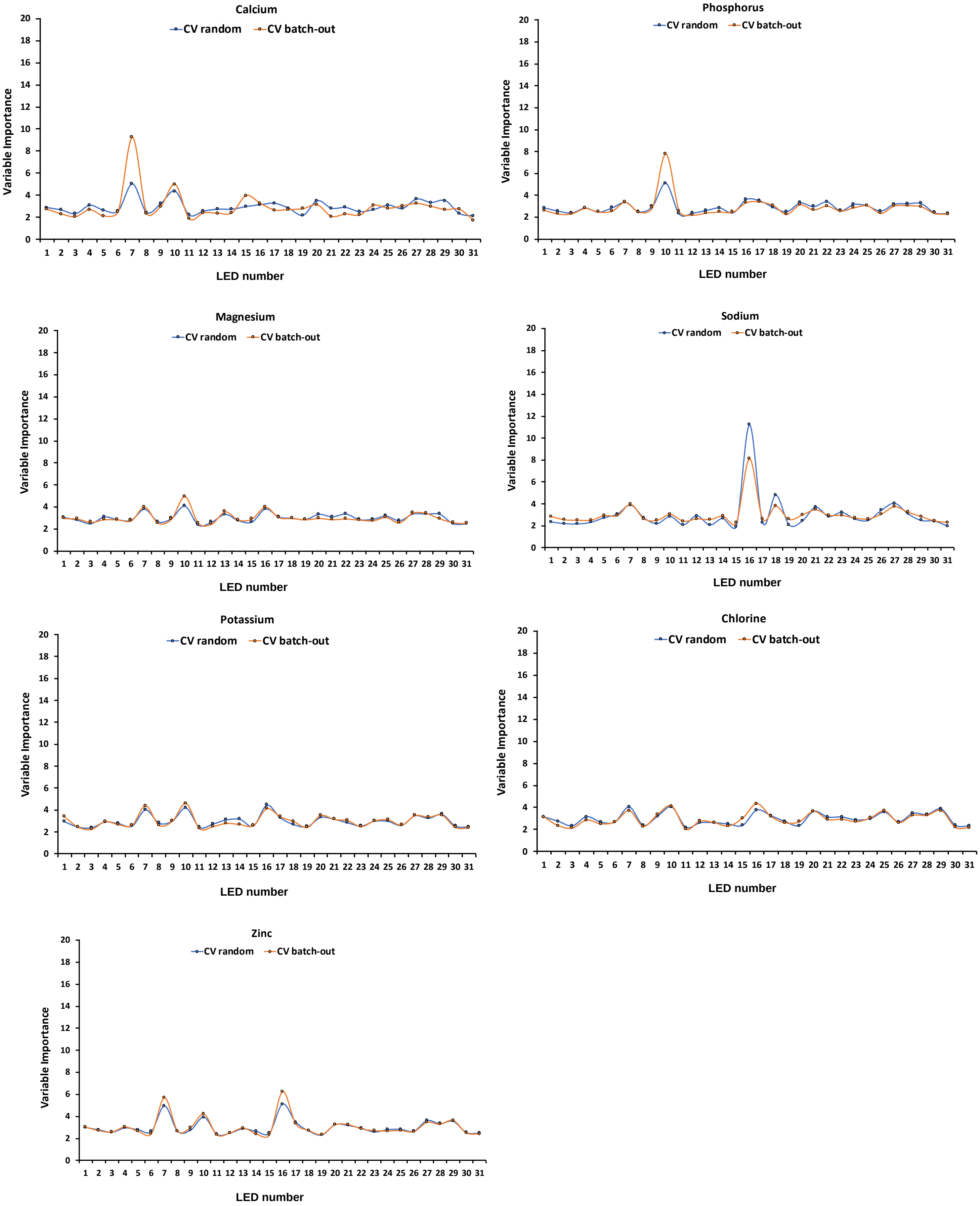

Supplementary Figure S6. Density distribution of hematochemical parameters. For hematocrit, non-esterified fatty acids (NEFA), β-Hydroxybutyric acid (BHBA), aspartate aminotransferase (AST), γ-glutamyl transferase (GGT), creatinine, total bilirubin (BILt), haptoglobin and ferric reducing antioxidant power (FRAP) log-10 transformation have been applied, and distribution before and after transformation is showed. CP = ceruloplasmin; ALP = alkaline phosphatase; MPO = myeloperoxidase; ROMt =total reactive oxygen metabolites; AOPP = advanced oxidation protein products; FRAP = ferric reducing antioxidant power; SHp = thiolic groups. Graphics have been created using the R software v. 3.6.3 (www.r-project.org).

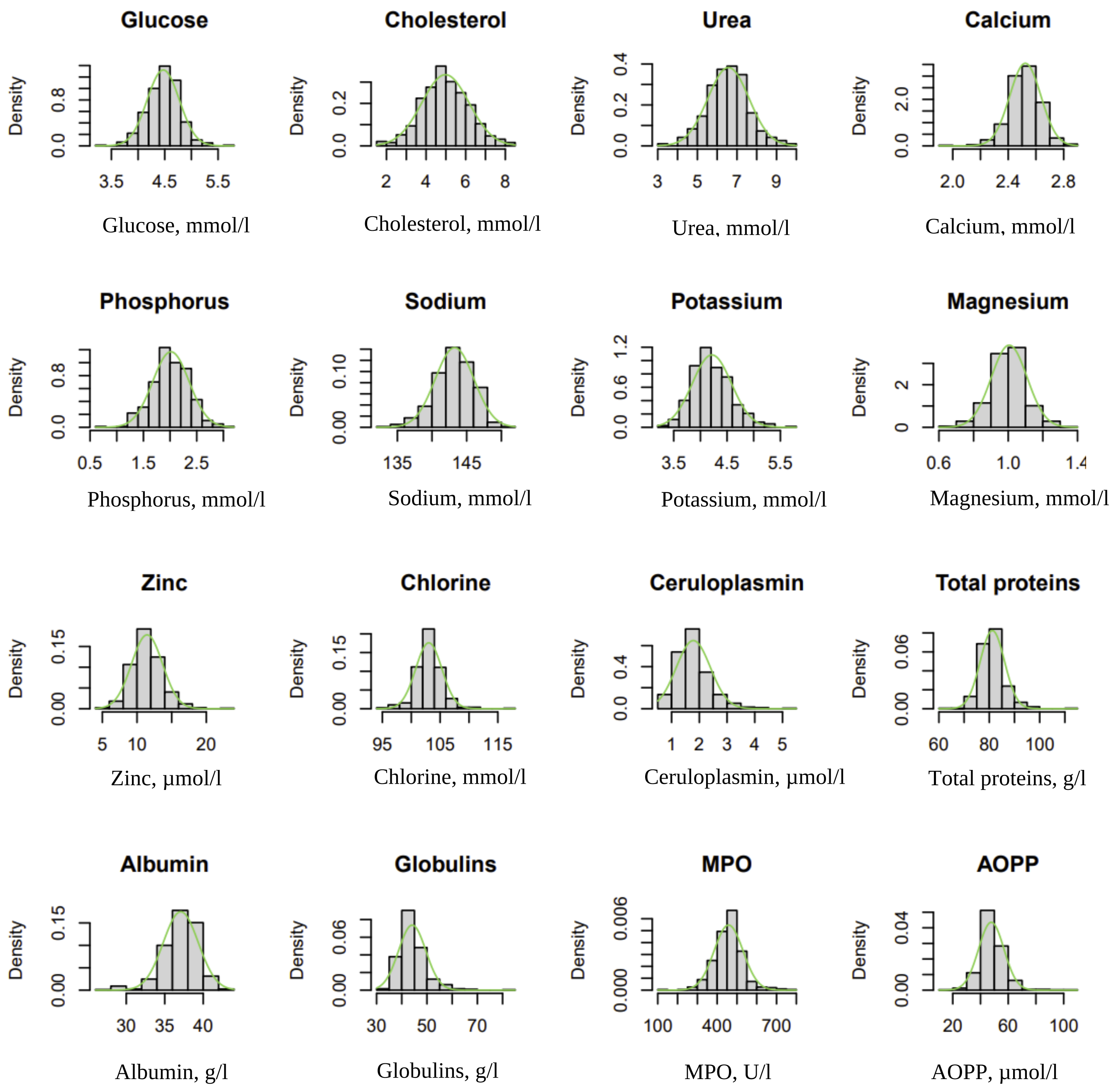

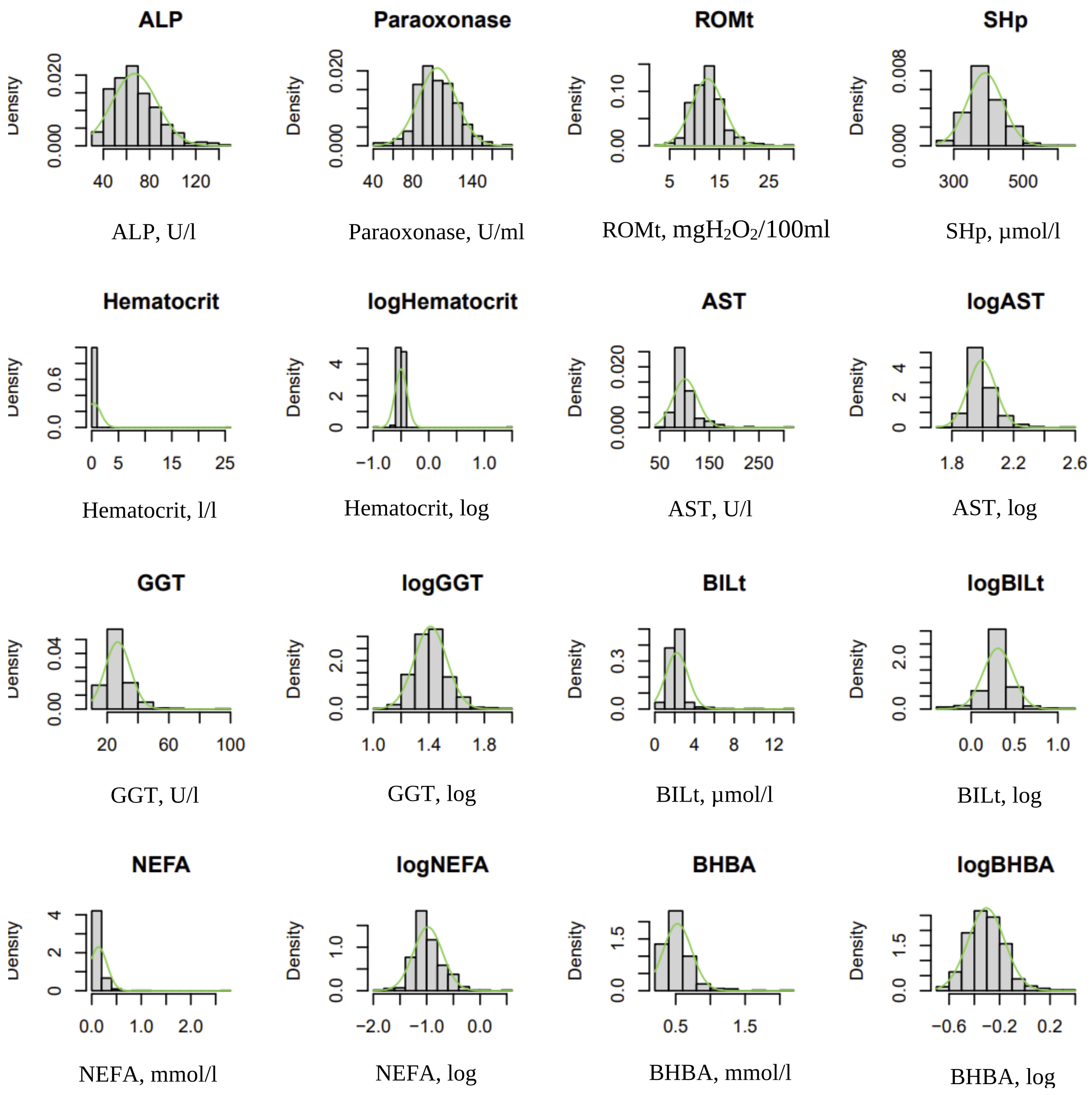
ALP
Density
0.000 0.020
40 80 120
ALP, U/l
Paraoxonase
Density
0.000 0.020
40 80 140
Paraoxonase, U/ml
ROMt
Density
0.00 0.10
5 15 25
ROMt, mgH$_2$O$_2$/100ml
SHp
Density
0.000 0.008
300 500
SHp, µmol/l
Hematocrit
Density
0.0 0.6
0 5 15 25
Hematocrit, l/l
logHematocrit
Density
0 2 4
−1.0 0.0 1.0
Hematocrit, log
AST
Density
0.000 0.020
50 150 250
AST, U/l
logAST
Density
0 2 4
1.8 2.2 2.6
AST, log
GGT
Density
0.00 0.04
20 60 100
GGT, U/l
logGGT
Density
0.0 2.0
1.0 1.4 1.8
GGT, log
BILt
Density
0.0 0.3
0 4 8 12
BILt, µmol/l
logBILt
Density
0.0 2.0
0.0 0.5 1.0
BILt, log
NEFA
Density
0 2 4
0.0 1.0 2.0
NEFA, mmol/l
logNEFA
Density
0.0 1.0
−2.0 −1.0 0.0
NEFA, log
BHBA
Density
0.0 1.5
0.5 1.5
BHBA, mmol/l
logBHBA
Density
0.0 1.5
−0.6 −0.2 0.2
BHBA, log

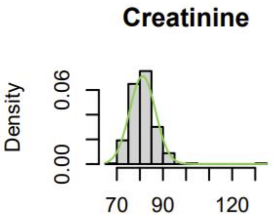

Creatinine, µmol/l

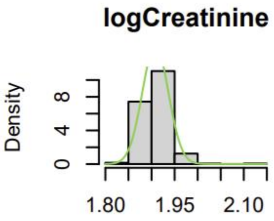

Creatinine, log

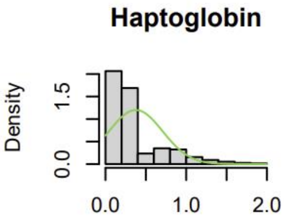

Haptoglobin, g/l

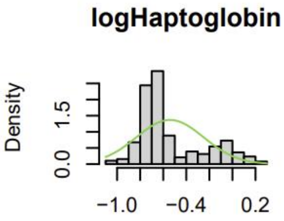

Haptoglobin, log

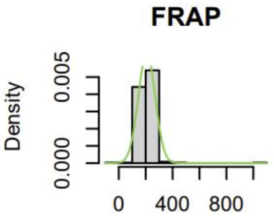

FRAP, µmol/l

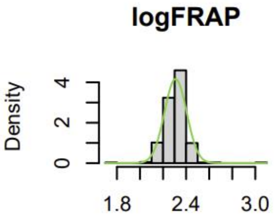

FRAP, log
